# Plasma Arc Robot for Direct Wall High-Entropy Alloy Additive Manufacturing

**DOI:** 10.3390/ma19020354

**Published:** 2026-01-15

**Authors:** Wei Wu, Haoran Wang, Yani Hu, Yan Lu, Jietao She, Xianghui Ren

**Affiliations:** 1 School of Automobile and Transportation Engineering, Guangdong Polytechnic Normal University, Guangzhou 510665, China; wuwei_5v@126.com (W.W.); whr54281015@163.com (H.W.);; 2 Yangjiang China-Ukrainian E. O. Paton Institute of Technology, Yangjiang 529533, China; 3 China-Ukrainian Institute of Welding, Guangdong Academy of Science, Guangzhou 510650, China

**Keywords:** plasma arc, high-entropy alloy, microstructure, mechanical properties

## Abstract

**Highlights:**

**What are the implications of the main findings?**

- Thin walled AlCoCrFeNi high-entropy alloy was prepared using plasma arc additive manufacturing combined with swing technology to improve forming quality and material density.
- Systematically study the microstructural evolution and anisotropic mechanical properties, and reveal the mechanism of the influence of sedimentary height on the evolution of FCC/BCC phases.

**Abstract:**

Through the mechanical analysis of AlCoCrFeNi thin-walled high-entropy alloy materials fabricated by plasma arc additive manufacturing, this study examines the practical application prospects of plasma arc manufacturing technology for thin-walled high-entropy alloys and explores its future development directions. Using a plasma arc oscillation process, a 50-layer fine additive experiment was conducted on AlCoCrFeNi high-entropy alloy materials employing both reciprocating and layer-by-layer accumulation methods. The samples were analyzed for overall appearance, microstructure, hardness, and tensile properties. The results indicate that the proportions of columnar and intergranular dendrites in the thin-walled high-entropy alloy specimens are similar, and the columnar dendrites exhibit a uniformly sized cross shape. The variation in Vickers microhardness along the horizontal direction shows lower strength at the edge positions, gradually increasing with horizontal distance. A comparison of the alloy’s transverse and longitudinal tensile specimens revealed that samples parallel to the deposition direction exhibit more regular structural arrangements, while specimens perpendicular to the deposition direction show unavoidable stress concentration at the deposition sites during tensile testing. With the increase in the height of the longitudinal specimens, the FCC structures in the alloy are significantly refined, the organizational arrangement becomes more regular, and the elongation increases. This study elucidates the plasma arc preparation technique for thin-walled high-entropy alloy materials, which is expected to achieve precise control over material composition, accurate observation of grain refinement, and uniform distribution of Vickers hardness, thereby enhancing the mechanical properties and thermal stability of the materials, with promising applications in aerospace, energy, and industrial fields.

## 1. Introduction

With the advancement of technology, traditional alloys and conventional preparation methods increasingly struggle to meet diverse demands. As a new-generation product, high-entropy alloys (HEAs) are mixtures composed of five or more metallic elements with similar molar fractions for each component. Unlike traditional alloys, which typically feature a single matrix element, HEAs involve interactions among multiple principal elements, enabling them to exhibit composite effects in performance [1]. While HEAs hold significant potential in major engineering fields, issues such as high costs persist. Consequently, research on HEAs remains largely theoretical, with limited practical application. The development of low-cost, high-performance HEAs will be a key focus in future research [2].

In high-entropy alloys, the atomic radii and electronegativity differences among transition elements such as Co, Cr, Fe, and Ni are relatively small, and the mixing enthalpy between these elements approaches zero, making them prone to forming FCC-type substitutional solid solutions. Consequently, research on CoCrFeNi-based high-entropy alloys has been extensive. CoCrFeNi high-entropy alloys exhibit minimal lattice distortion, offering good ductility and plasticity but relatively low strength. Adding Al is the most common method to enhance the hardness and strength of CoCrFeNi high-entropy alloys [3]. Currently, the most widely studied dual-phase high-entropy alloy systems include the FeCoNiCrAl_x_ series with face-centered cubic (FCC) + body-centered cubic (BCC) structures [4]. These alloys demonstrate excellent ductility, high-temperature stability, oxidation resistance, and electromagnetic properties, making them promising candidates to replace nickel-based superalloys in applications such as nuclear energy and aerospace [5,6]. In addition, the solubility of high-entropy alloys must be considered. The solubility of high-entropy alloys is essentially the result of the combined effects of thermodynamics (high-entropy effect) and kinetics (diffusion and cooling). By optimizing the composition design and process parameters, an efficient solid solution of elements can be achieved, thereby obtaining alloy materials with high strength and stability.

However, traditional high-entropy alloy materials are primarily prepared through powder metallurgy or melting methods. Conventional as-cast AlCoCrFeNi-based high-entropy alloys generally suffer from issues such as low strength, poor wear resistance, and corrosion resistance [2]. Researchers have found that altering the preparation method of AlCoCrFeNi can enhance its performance [7,8].

Therefore, investigating the enhancement pathways of the mechanical properties of Al_x_CoCrFeNi high-entropy alloys is of great significance for the industrial application of high-entropy alloys and advanced mechanical manufacturing.

Additive manufacturing (AM) excels in producing complex and customized geometries that are difficult to achieve with traditional manufacturing methods. By minimizing waste, improving material utilization, reducing costs, and decreasing environmental impact, these advantages make it highly promising for applications across industries such as aerospace, healthcare, and automotive manufacturing [9,10].

Moreover, AM supports multi-material manufacturing, enabling the integration of materials with diverse properties in a single build. Consequently, some scholars have employed additive manufacturing techniques such as laser melting and selective laser sintering (SLM) to investigate the microstructural evolution and mechanical properties of Al_x_CoCrFeNi high-entropy alloys. For instance, Yu et al. [11] successfully fabricated CoCrFeNi high-entropy alloys with varying Al contents via laser metal deposition, examining the effects of increasing Al content on microstructure and performance, as well as their high-temperature properties and oxidation behavior. Another study demonstrated that adding aluminum could effectively optimize the phase structure of the alloy, enhancing its strength and corrosion resistance [12,13]. Additionally, Qiu et al. [14] and Chen [15] separately produced AlCoCrFeNi high-entropy alloys using selective laser melting technology, studying the phase distribution, microstructure, and strengthening mechanisms of AlCoCrFeNi high-entropy alloys, as well as the influence of different process parameters on the cladding quality of AlCoCrFeNi high-entropy alloys. Meanwhile, Yao et al. [16] utilized twin-wire arc additive manufacturing to produce AlCoCrFeNi-based high-entropy alloys and conducted research on their microstructure and properties.

These studies indicate that the combination of additive manufacturing and composition adjustment can effectively enhance the performance of Al_x_CoCrFeNi high-entropy alloys, promoting the development of this material in high-performance applications.

Plasma arc manufacturing technology (PAAM) has a higher material utilization rate compared to other additive manufacturing methods [17], especially suitable for manufacturing high-melting-point metals and alloys [18]. In addition, PAAM has a relatively low heat input, which can reduce thermal stress and residual stress, and improve the comprehensive mechanical properties of workpieces [19]. Therefore, plasma arc technology can effectively prepare high-entropy alloy materials, and by optimizing plasma arc manufacturing parameters, the performance of the materials can be further improved. Wang [20] studied the effect of plasma arc additive manufacturing on the intermetallic compounds of CoCrFeNiSi_x_ high-entropy alloys at different Si element molar ratios. Yang [21] employed plasma arc wire additive manufacturing technology to study the Ti-5Al-6Mo-3Cr-0.1B alloy and analyzed the effects of different heat treatment regimes on the alloy’s mechanical properties and microstructural morphology. Nguyen et al. [22] employed a novel spot welding technique using a dual-nozzle welding gun and utilized a stabilized plasma arc to develop a welding process for ultra-thin metal sheets, studying its efficiency improvement, quality control, and heat input reduction characteristics. Zhang et al. [23] prepared Al_0.3_CoCrFeNi high-entropy alloy using directional solidification technology and investigated the relationship between the microstructure evolution and mechanical properties of the alloy under directional solidification. The results indicate that the microstructure of the alloy after directional solidification treatment transforms from dendritic crystals to columnar crystals or even single crystals. The fracture strength of the alloy has been significantly improved, with an 80% increase in strength compared to the alloy obtained by ordinary casting. Therefore, the use of arc additive manufacturing technology can achieve directional solidification of high-entropy alloys.

It can be seen that previous studies have largely focused on the research of different elemental contents in high-entropy alloys and the effects of these varying elemental contents on intermetallic compounds. In summary, this paper uses AlCoCrFeNi alloy powder as the primary raw material. After pretreatment, additive raw material is obtained. During the preparation process, the raw material is gathered at the nozzle through a powder feeding device and, combined with the high temperature of the plasma arc, is melted and re-solidified to form the cladding layer, achieving a 50-layer additive experiment. The paper investigates the overall appearance of the samples as well as the microstructure and mechanical properties of various parts, providing a foundation for the subsequent production of high-quality, high-entropy alloy materials.

## 2. Materials and Methods

### 2.1. Experimental Materials

This experiment uses PSPW-AICo1 brand AlCoCrFeNi alloy powder as the main raw material, with a purity of (100 wt.%) cobalt, chromium, iron, nickel, and aluminum, and powder particle diameters ranging from 45 to 150 μm. The results of analyzing the alloy composition using MESA-50 X-ray fluorescence spectrometer (Thermo Fisher Scientific, Waltham, MA, USA) are shown in Table 1. There is a certain deviation between the actual content and nominal composition of various elements. According to the mass percentage, the specific alloy composition ratio of thin-walled high-entropy alloy material is calculated and shown in Table 1. According to the molar ratio and specific gravity of the components, the high-entropy alloy material selected in this experiment is AlCoCrFeNi2.

The substrate is made of a 250 mm × 200 mm × 5 mm 316 L stainless steel plate. Before the test, the surface of the substrate was polished and cleaned with acetone and dried. We used argon gas with a purity of 99% as the protective gas and ion source. The additive manufacturing test system consists of a PMI-350AC/DC TL plasma AC-DC inverter welding machine (Panasonic Industry Europe GmbH, Lüneburg, Germany), AFS-PF-F multi barrel synchronous powder feeder, and KUKA 6-axis welding robot. This experiment employs a plasma arc oscillation process, using robotic reciprocation and layer-by-layer deposition, to perform 50-layer cladding of high-entropy alloy materials through a zigzag scanning pattern. The distance between the welding gun and the substrate is 8 mm. After each layer is welded with a length of 140 mm, the welding gun is lifted 2 mm to deposit the next layer. Other parameters were as follows: plasma deposition current of 120 A, protective gas and ion gas flow rates of 15 and 1.5 L/min, powder feeding speed of 2 RPM, powder feeding gas flow rate of 350 L/h, interlayer cooling temperature of 80 °C, and walking speed of 3 mm/s.

### 2.2. Preparation Method of Test Samples

**Figure 1:**
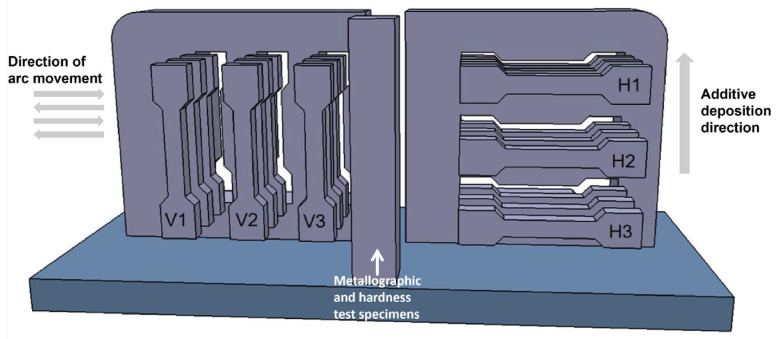
Schematic diagram of sampling thin-walled high-entropy alloy deposition samples.

**Figure 2:**
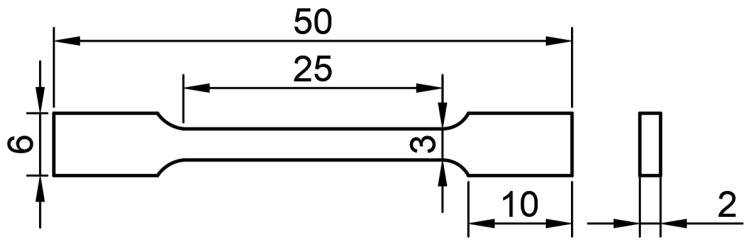
Dimensional drawing of tensile test piece (mm).

After the additive manufacturing experiment is completed, metallographic specimens and tensile test pieces are taken from the samples using the wire-cutting method for performance characterization. We selected longitudinal tensile samples V1~V3 in the left area of the thin-walled sedimentary specimen, and cut 3 samples in the thickness direction at each position for tensile testing to take the average value. We selected transverse tensile samples H1~H3 in the right area, and similarly cut 3 samples in the thickness direction at each position. We took metallographic and hardness samples from the middle, as shown in Figure 1. We pretreated the metallographic samples by grinding them with SiC sandpaper of 400 #, 800 #, and 1200 # in sequence, followed by polishing with W0.25 diamond grinding paste on a polishing machine, and then cleaning and drying with alcohol. Subsequently, the polished surface of the sample was corroded in a mixed solution containing 75% HCl and 25% HNO_3_, and the evolution process of the metallographic structure in the deposition direction was observed and analyzed using the NOVA Nano Scanning Electron Microscope SEM430 (FEI Company, Hillsboro, OR, USA). Using the HMV-2T Micro Vickers Hardness Tester (Shimadzu, Kyoto, Japan), the hardness of the specimen was measured every 2 mm along the deposition direction in the middle of the cross-section under a load of 500 g and a holding time of 10 s. In order to comprehensively evaluate the mechanical properties of thin-walled sedimentary specimens, the GP-TS2000M/300KN universal tensile testing machine (Shenzhen Gaopin Testing Equipment Co., Ltd., Shenzhen, China) was used to conduct tensile performance tests on the specimens at different positions at room temperature according to the standard GB/T228.1-2021 [24]. The dimensions of the specimens are shown in Figure 2.

## 3. Results and Analysis

### 3.1. Macroscopic Morphology

**Figure 3:**
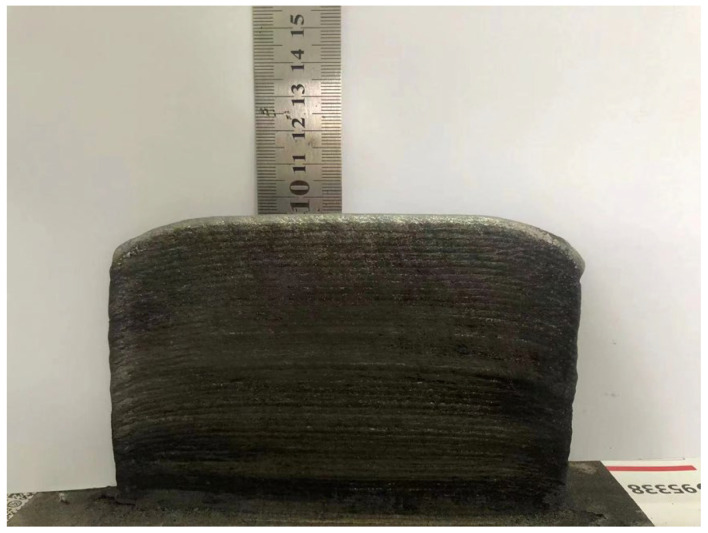
Macroscopic appearance of the sample.

The overall view of the 50-layer sample deposited with high-entropy alloy is shown in Figure 3. The clear and visible interlayer boundaries indicate a stable sedimentary process. The total length of the sample is 140 mm, with an average height of 94 ± 0.1 mm. Due to the deposition using a reciprocating welding gun, the heat dissipation time at both ends of the deposition is the shortest, resulting in severe heat accumulation and significantly lower heights on both sides of the sample compared to the middle. During the experimental deposition process, there were no obvious macroscopic defects such as lack of fusion or cracks observed on the surface of the sample. The high temperature of the plasma arc power supply enabled the powder to fully melt, which reduces defects such as porosity and lack of fusion [25].

### 3.2. Microstructure

**Figure 4:**
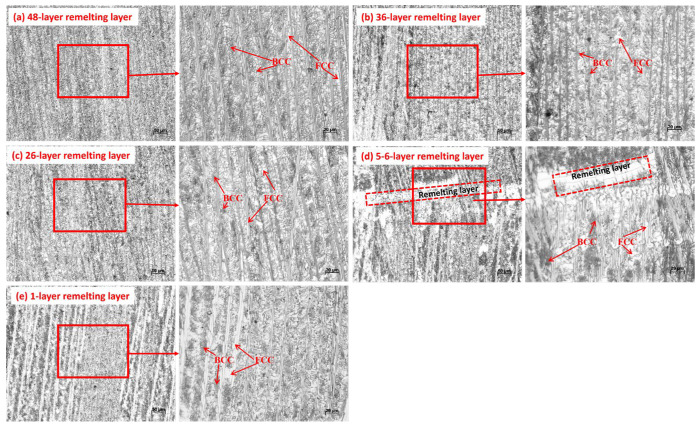
Metallographic diagram of different cross-sectional layers of the sample.

Figure 4 shows the microstructure of AlCoCrFeNi_2_ high-entropy alloy thin walls prepared by the plasma arc method at different positions. According to the microstructure diagram, it can be seen that the microstructure of the AlCoCrFeNi_2_ thin-walled high-entropy alloy prepared is dendritic structure, with similar proportions of crystal, dry and intergranular, uniform distribution, and clear boundaries. The dendritic dry mainly appears as long strips. During the high-temperature heating and cooling process of plasma arc, i.e., eutectic reaction, a clear two-phase network structure was observed, and the alloy was a FCC + BCC dual-phase structure mainly composed of a BCC (body-centered cubic) phase [26]. In high-entropy alloy systems, the free energy is relatively low, and the thermodynamic significant mixing entropy effect can effectively suppress the formation of intermetallic compounds, making the alloy easy to form simple solid solution phases such as FCC face centered cubic structures [25] and BCC body-centered cubic structures [27,28]. During the plasma arc metallurgy process of AlCoCrFeNi_2_ powder, high melting point elements Fe and Cr nucleate and form dendrites from the liquid. Heating during the deposition process promotes dendrite growth, while Ni element is eliminated by the FCC structure phase. Al and remaining Ni are separated again [29], ultimately forming a new phase with the BCC structure [2]. The arrows in the figure indicate alternating eutectic layered structures, with the white matrix consisting of FCC phases rich in Fe, Co, and Cr, and the black irregular block/needle shaped parts consisting of BCC phases rich in Al and Ni [30,31,32].

During the melting and deposition process, due to the high thermal conductivity of the bottom layer, the dominant heat flow direction of the metal in the molten pool is perpendicular to the plasma arc scanning direction and transmitted to the bottom, so the solidification process moves from the bottom to the top of the molten pool [33]. The remelting lines between the sedimentary layers are clearly visible, and the dendritic structure in the alloy prepared by plasma arc layer by layer deposition presents columnar crystals with epitaxial growth, as shown in Figure 4d. The columnar grains are approximately perpendicular to the scanning trajectory direction, because the heat generated by the plasma arc causes rapid heating and cooling of the melt pool. According to the solid-phase transition theory, the growth direction of grains is usually along the temperature gradient direction, so the columnar grains usually grow towards the cooling direction, forming an orientation perpendicular to the scanning trajectory. The deposition direction also determines the direction of heat dissipation, ultimately leading to preferential growth of grains along the deposition direction. Due to the large temperature gradient between the bottom of each cladding layer and the top of the previous layer, the heat dissipation direction is perpendicular to the “substrate” of each layer, resulting in columnar crystals perpendicular to the scanning trajectory [34].

As the additive height increases, the AlCoCrFeNi_2_ thin-walled high-entropy alloy undergoes different thermal cycles in each region, resulting in differences in heat accumulation and solidification cooling, leading to differences in the microstructure of each part. Fe and Cr with a molar ratio close to 1:1 are enriched in the intergranular region [35,36].

Eutectic structures have precipitated between the lower grains. Due to the coverage of the upper layer material, the heat dissipation rate in the lower region is relatively slow, which provides more time for the precipitation of eutectic structures and makes it easier for the grain boundaries to widen [37], resulting in the boundary between grains and crystal dryness. From Figure 4e, it can be seen that the bottom of the sedimentary thin wall is composed of a coarse columnar dendritic structure and irregular cellular dendritic structure. This is mainly attributed to the good thermal conductivity of the substrate, which facilitates the transfer of heat during the solidification of the interlayer melt pool, and is conducive to the formation and growth of a coarse columnar dendritic structure [38]. In addition, the bottom has the most heating cycles, and FCC, as a high-temperature stable phase, provides the driving force for the migration of Al and Ni elements at high temperatures after multiple thermal cycles [39]. As the sedimentation height increases, the dendritic structure in the alloy undergoes significant refinement, and the organization arrangement in the alloy becomes more regular. From the figure, it can be seen that the structure of the alloy is clear and dense, without any serious defects. The proportion of dendrites with black stripe morphology significantly increases in AlCoCrFeNi_2_ thin-walled high-entropy alloy. This is mainly due to the thermal accumulation effect of layer-by-layer deposition, which allows sufficient time for dendritic growth, and its rapid solidification is conducive to the formation of the B2 phase [40].

The central region of Figure 4b,c is mainly composed of columnar and fine-layered structures, accompanied by a small amount of thicker and longer-layered structures. Due to the fewer thermal cycles compared to the bottom region, the FCC phase content is lower in the bottom region. The upper region of Figure 4a has the lowest number of thermal cycles, and compared to the bottom and middle regions, more FCC phases are transformed into BCC phases, presenting a finer layered structure.

### 3.3. Hardness Analysis

**Figure 5:**
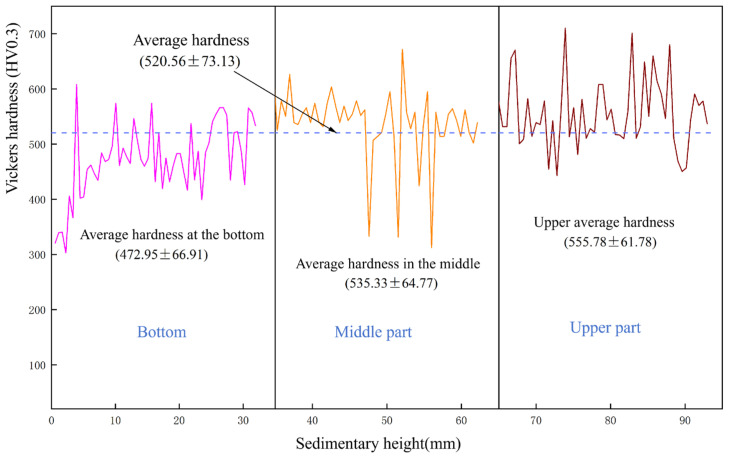
Hardness curve of the sample as a function of sedimentation height.

Figure 5 shows the Vickers microhardness test curve results of the AlCoCrFeNi2 high-entropy alloy cross-section from bottom to top. It was observed that as the deposition height of the sample increased, the Vickers microhardness of the thin-walled high-entropy alloy also increased. The average hardness of the bottom, middle, and upper parts is 472.95 ± 66.91 HV0.3, 535.33 ± 64.77 HV0.3, and 555.78 ± 61.78 HV0.3, respectively. This is consistent with the results of microstructure analysis. As the deposition height increases, the grain size gradually decreases and the dendrite spacing also decreases. This grain refinement phenomenon leads to the so-called fine grain strengthening effect, where the decrease in grain size increases the Vickers microhardness of the material [41,42].

In addition, Vickers microhardness is closely related to the formation of FCC and BCC structures in high-entropy alloys. The elemental composition characteristics of high-entropy alloys make them more prone to forming an FCC structure, where atoms occupy most of the lattice positions according to the FCC arrangement, resulting in a stable FCC structure. As defect-free layers accumulate gradually, heat accumulates between the cladding layers, causing an increase in interlayer temperature and a gradual rise in BCC content, thereby enhancing the material’s Vickers microhardness. In the bottom region, a BCC → FCC phase transformation occurs, with the FCC phase being more abundant than in the middle and top regions, leading to lower hardness in the bottom region compared to the middle and top regions. The FCC phase content in AlCoCrFeNi_x_ as cast high-entropy alloy increases with the increase in Ni content [29]. The high-Ni-based high-entropy alloy used in this article provides favorable conditions for the formation of a FCC phase, thereby significantly improving the overall hardness of the sample. The average Vickers microhardness of the high-entropy alloy obtained through plasma arc additive manufacturing is 520.56 ± 73.13 HV0.3, indicating that this manufacturing method and process can achieve high-hardness, high-entropy alloys. The unevenness of hardness distribution indicates that the material has undergone complex heat treatment processes during the deposition process, which has a significant impact on the final properties of the material.

### 3.4. Tensile Test

Compared to BCC, FCC has more slip systems and higher crystal structure symmetry, which endow it with good plasticity and toughness. In AlCoCrFeNi high alloy, the FCC phase is distributed in a grid-like pattern, providing excellent plasticity and toughness for the alloy. The BCC phase with submicron amplitude modulation structure significantly enhances the strength of the alloy [43,44].

Based on the tensile results of six samples of AlCoCrFeNi2 thin-walled high-entropy alloy presented in Table 2, the performance differences between transverse tensile specimens (H1, H2, H3) and longitudinal tensile specimens (V1, V2, V3) were compared. The results showed that the longitudinal tensile specimens parallel to the additive deposition direction (V series) were significantly better than the transverse tensile specimens perpendicular to the deposition direction (H series). This difference is mainly attributed to the fact that the longitudinal specimens are parallel to the additive deposition direction, resulting in a more regular arrangement of the alloy structure. However, the transverse specimen exhibits stress concentration at the deposition site and shows significant mechanical anisotropy [45].

For the vertically stretched samples (V series), there is not much difference in their performance. The average values of maximum tensile strength, yield strength, and elongation are 2092 ± 51.4 MPa, 1201.3 ± 52.8 MPa, and 6.8 ± 1.7%, respectively. This is mainly because the vertical samples are taken from the same layer position of the deposited samples, and their properties are similar, which also reflects the stability of the deposition process and the uniform melting of powder metallurgy. It is worth noting that although the maximum tensile strength of V3 is slightly higher than that of V1 and V2, this may be due to a slight strengthening effect caused by differences in speed control during tensile testing, as all specimens are derived from the same sample with similar raw materials and structures.

In contrast, the average maximum tensile strength, yield strength, and elongation of the horizontally stretched samples (H series) are 1680.3 ± 157.9 MPa, 1218.7 ± 60.5 MPa, and 12 ± 0.06%, respectively. Due to the selection of stretching in the upper, middle, and lower parts, there are significant differences in the performance of each part. As the height of the sample increases, layer-by-layer accumulation leads to an increase in temperature, and the BCC content gradually increases, thereby improving hardness and tensile strength [25]. The average maximum tensile strength of H3 is 1900 ± 31.5 MPa, which is 23.7% higher than H1 (1536 ± 25.2 MPa), consistent with the results of microstructure and hardness analysis. In addition, the elongation of vertical tensile samples (V series) is generally higher than that of horizontal tensile samples (H series). This improvement is attributed to the columnar crystals grown epitaxially in the arc additive manufacturing process, which have greater strength and plasticity in the longitudinal direction [46]. At the same time, the difference in yield strength in the table is not significant, and the rigidity and plasticity of the material are similar, indicating that the material prepared by this process has similar resistance to deformation and does not have anisotropy.

**Figure 6:**
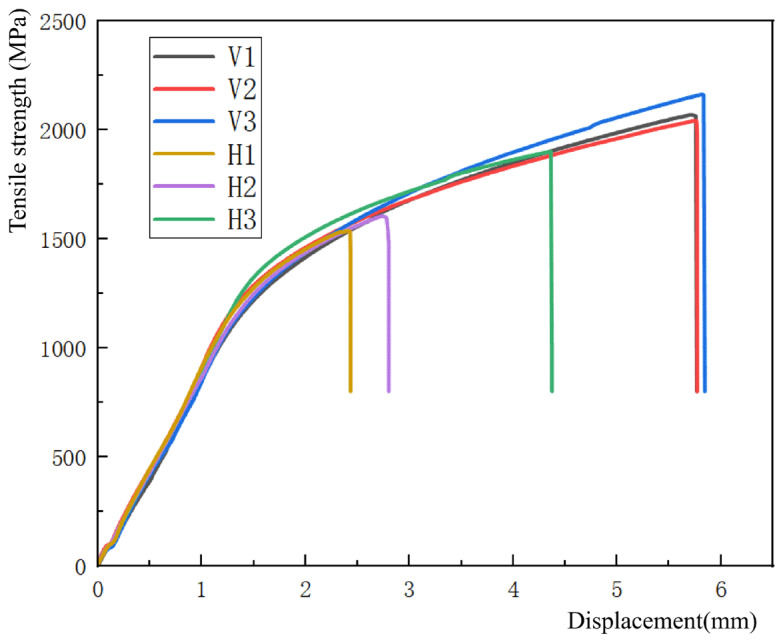
Strength displacement curve of each specimen.

Figure 6 visually displays the tensile strength displacement curves of six tensile specimens, further verifying the differences in performance mentioned above. The trend of elongation variation also indicates that the elongation of vertical tensile samples (V series) is generally higher than that of horizontal tensile samples (H series), with a maximum elongation of 12.072 ± 0.8% for V1 and a minimum elongation of 5.25 ± 0.09% for horizontal sample H3. This difference is mainly due to significant changes in the FCC microstructure of the upper and lower parts of the alloy. The FCC structure significantly improves the toughness of the alloy [47,48], while the BCC phase mainly enhances the strength and hardness of the alloy. The presence of a large amount of BCC phase deteriorates the plasticity of the alloy [30]. Therefore, the plasticity of the upper horizontally stretched sample is poor.

Based on the comprehensive evaluation of the tensile performance, it can be seen from the strength plastic product data presented in Table 2 that the maximum value is approximately 25.8 GPa%, which is equivalent to the performance of the CoCrNi high-entropy alloy studied by Xu Ning [49]. Therefore, thin-walled high-entropy alloy samples with good comprehensive mechanical properties can be prepared through plasma arc additive manufacturing technology.

## 4. Conclusions

1. Plasma arc manufacturing technology has prepared thin-walled high-entropy alloy materials, and the alloy structure of the obtained samples is clear and dense, with no serious defects in the alloy.
2. The microhardness of thin-walled high-entropy alloy materials changes along the horizontal position, and it can be seen that the strength of thin-walled high-entropy alloy materials is lower at the edge position, showing an upward trend with the increase in horizontal distance.
3. The microstructure of the prepared thin-walled high-entropy alloy is a dendritic structure, with boundaries between grains. As the height increases, the organization arrangement in the alloy becomes more regular. A small amount of Cr reduces the grain size. The distribution of Fe, Co, and Ni elements in the alloy after the segregation weakening treatment is uniform. The segregation phenomenon of Al and Cr elements in the alloy is enhanced.
4. The average maximum tensile strength in longitudinal tensile specimens is 783 ± 121 MPa, while in transverse tensile specimens, the equilibrium maximum tensile strength of the alloy is 1122 ± 589.2 MPa. The compressive strength of the alloy increases by 43.29%, indicating good tensile performance. This gradient material sample has high strength and toughness, and can meet various industrial needs.

In summary, the future direction of preparing straight-arm high-entropy alloys using plasma arc technology will focus on three core aspects: adaptability to extreme environments, process economy, and functionality of microstructures. Through material innovation and technological breakthroughs, it aims to advance from the laboratory to industrial applications in strategic fields such as aerospace, nuclear energy, and energy sectors.

## Figures and Tables

**Table 1:** Chemical element content and molar mass of AlCoCrFeNi powder.

Elements	Calibration Components (wt.%)	Actual Measured Components (wt.%)	Molar Mass (g/mol)
Al	8.51	8.35	27
Cr	18.59	17.27	52
Co	17.62	18.70	58.933
Fe	16.40	18.11	56
Ni	38.88	37.57	58.69

**Table 2:** Tensile properties of the sample.

Samples	Maximum Tensile Strength (MPa)	Yield Strength (MPa)	Elongation (%)	Strong Plastic Accumulation (GPa%)
H1	1536 ± 25.2	1170 ± 18.8	9.156 ± 0.3	14.1
H2	1605 ± 16.3	1182 ± 16.1	6.032 ± 0.2	9.7
H3	1900 ± 31.5	1304 ± 8.8	5.25 ± 0.09	10.0
V1	2070 ± 11.9	1162 ± 23.2	12.072 ± 0.8	25.0
V2	2043 ± 8.7	1166 ± 12.8	11.954 ± 0.7	24.4
V3	2163 ± 20.5	1276 ± 21.3	11.946 ± 0.9	25.8

## Data Availability

The original contributions presented in this study are included in the article. Further inquiries can be directed to the corresponding author.
